# Barriers and facilitators of adherence to the use of ASICA, a digital app designed to support people previously treated for melanoma: concise report of a qualitative study

**DOI:** 10.1093/ced/llad279

**Published:** 2023-08-23

**Authors:** Maria Ntessalen, Sajan McCorkindale, Albana Krasniqi, Heather M Morgan, Julia L Allan, Peter Murchie

**Affiliations:** Institute of Applied Health Sciences, Academic Primary Care, Polwarth Building, Aberdeen Scotland, UK; Institute of Applied Health Sciences, Academic Primary Care, Polwarth Building, Aberdeen Scotland, UK; Institute of Applied Health Sciences, Academic Primary Care, Polwarth Building, Aberdeen Scotland, UK; Institute of Applied Health Sciences, Academic Primary Care, Polwarth Building, Aberdeen Scotland, UK; Institute of Applied Health Sciences, Academic Primary Care, Polwarth Building, Aberdeen Scotland, UK; Institute of Applied Health Sciences, Academic Primary Care, Polwarth Building, Aberdeen Scotland, UK

## Abstract

We developed the Achieving Self-directed Integrated Cancer Aftercare (ASICA) in melanoma app to support monthly total-skin self-examinations (TSSE) by people previously treated for melanoma. A randomized 12-month trial demonstrated ASICA supported optimal monthly TSSE adherence in a third of participants (ClinicalTrials.gov NCT03328247). However, a further third of participants adhered well initially but subsequently dropped off, and a final third did not adhere at all. This follow-up qualitative study investigated trial participants’ perceptions of barriers and facilitators to TSSE adherence using the app. Three former trial participants participated in a single focus group and 11 participated in new semistructured telephone interviews. These were analysed thematically alongside secondary analysis of 13 qualitative interviews conducted during the trial. All transcripts were recorded, transcribed and analysed thematically. Five themes encompassing barriers and facilitators to ASICA adherence emerged. These were: technology, role of others, tailoring, disease journey and competing priorities. These data will inform further development of ASICA to increase user adherence.

## Teaser

Digital apps are used to manage diseases but adherence to them is still not optimal. We explored the opinions of patients with melanoma on how we can improve adherence to these apps. High levels of patient adherence and longer periods of use will benefit the patient by increasing their chances of better outcomes.

People previously treated for cutaneous melanoma are recommended to conduct monthly total-skin self-examinations (TSSEs) to enable the earliest detection of recurrence or new primaries, but only 20–25% report doing so.^1–3^ We developed the Achieving Self-directed Integrated Cancer Aftercare (ASICA) app with patients and clinicians to support monthly TSSE by people previously treated for melanoma.^4^ Briefly, ASICA is an app-based digital intervention prompting and guiding TSSEs and enabling users to send descriptions and photographs of any skin concerns to a remote dermatology nurse practitioner.

After refinement, ASICA was subject to a 12-month randomized controlled trial (ClinicalTrials.gov NCT03328247). Overall, the intervention group reported increased TSSE and improved quality of life.^5^ Most (57%) of the intervention group submitted details of a concern with most resolved without face-to-face consultation.^5,6^ We concluded that ASICA could support increased TSSE by people previously treated for melanoma, a view endorsed by a parallel qualitative study.^7^ Based on user feedback a ‘mocked-up’ smartphone (rather than the original tablet) version of ASICA was developed.

It has been observed, however, that digital healthcare adherence is subject to attrition where a proportion of users stop using the intervention over time, and nonadoption where users do not engage with the intervention at all.^8^ Adherence data from the ASICA trial supported this, identifying three distinct trajectories. An ‘adherent’ group of participants (41%) who consistently completed monthly TSSEs for 12 months; a ‘drop-off’ group of participants (36%) who adhered initially but with declining use over 2–6 months and a ‘nonadherent’ group of participants (24%) who failed to engage with the intervention despite enrolling.^9^ This qualitative study explored the perceptions of ASICA users of adherence barriers and facilitators.

## Report

Qualitative data derived from three sources. Previous trial participants participated in a facilitated focus group (3 attended) or semistructured telephone interviews (11 conducted) and were texted a link to a smartphone ‘mock-up’ of ASICA to view beforehand. The focus group lasted 98 min and interviews 14–58 min. We also conducted secondary analysis of 13 semistructured interviews (10–45 min) conducted with ASICA users to capture their experiences during the trial. These participants used the tablet version of ASICA and did not view the smartphone ‘mock-up’.^8^ The age range for the 27 participants was 29–86 years of age.

Transcripts were uploaded to NVIVO 1.6.1 and subject to thematic analysis.^10^ Five themes emerged: technology; role of others; tailoring; disease journey and competing priorities (see Appendix S1 in the Supporting Information for some illustrative quotes).

The first theme was technology. Several suggested the current interface had limitations that could hinder engagement and suggested improvements, especially developing a smartphone version. Some participants did not use technology routinely and others were unfamiliar with different operating systems. Our current prototype does not include artificial intelligence (AI); however, general concerns about emergent AI technology were viewed as a barrier to engaging with digital healthcare, particularly the robustness of diagnosis and a preference for human contact when worried. Conversely, one younger participant thought AI would enhance future adherence. One user expressed concerns about how personal data could be harvested while using digital healthcare. Other ideas to improve adherence included virtual small group sessions, an easy-to-navigate instructional video, and integrating social media functionality.

The second theme was the role of others. A few participants suggested engaging others would improve motivation and adherence. More than half were helped by other people to perform TSSEs, to check and photograph hard to see body areas or compare between current and previous skin images. Some participants said they were strongly motivated to adhere to the trial to help others in future. Several participants mentioned speaking to the dermatology nurse practitioner by phone and thought this subsequently sustained their adherence.

The third theme was tailoring. Participants thought adherence could improve if the app provided individually salient information but recognized that different people would want different things. Some felt a reminder of why skin checking was important would help. Other participants suggested news about research, information about skin protection products and ultraviolet weather reports would provide motivation. Tailoring monthly prompts and the importance of a reminder that worked for individuals featured prominently. Phone calls, email, autosyncing with calendars, an app-based prompt like food delivery apps, even a physical postcard, were all mentioned. It was also suggested that a prompt could arrive at the wrong time for participants (for example during work, on holiday). One participant felt ­regular prompts may be off-putting for some users and suggested individuals should be able to set their own schedule of TSSEs.

The fourth theme was disease journey. A third of participants suggested that adherence would likely drop as skin checking became less salient as time went on. They suggested that individuals may have heightened concern in the first few months but would accommodate to their condition over time and feel less need to check. It was noted that ongoing traditional face-to-face follow-up contributed to this process. On the other hand, it was acknowledged that individuals differed and that some may be motivated by ongoing concerns about recurrence, the belief that one annual check was insufficient and a sense of wellbeing produced by having completed the skin check.

The final theme was competing priorities Two-thirds of the participants noted that busy lives, for example work commitments affected adherence. They mentioned that other things take precedence and reduce the priority of skin checking. One participant had disengaged with TSSE during a bereavement. The burden of the task (i.e. having more moles to check) also contributed to disengagement.

##  

Digital technology can support regular TSSEs for people previously treated for melanoma, but more effectively for some than others. Our study sheds light on practical reasons why some people adhered more closely than others with corresponding implications for modification and tailoring. Our data, derived from 27 individuals at different stages of experience highlights several themes with the potential to be addressed in future, for example by simplifying the interface; introducing additional tailoring; encouraging engagement with others; and/or integrating smart scheduling. Participants who were socially deprived were underrepresented in the current sample, but urban and rural dwellers were included. Similarly, the sample included individuals from the adherent and drop-off groups but not any from the nonadherent group. Including individuals from the nonadherent group and other demographics, such as older people will be essential in future research to ensure digital healthcare does not increase health inequalities.

Overall, the information we have obtained from this study, in context with our other data, will inform the detailed modifications we are now making to ASICA.

## Learning points

- Achieving Self-directed Integrated Cancer Aftercare (ASICA), an app-based digital intervention increased regular total-skin self-examination and improved quality of life in people previously treated for melanoma.
- However, adherence varied with a third who adhered optimally but with adherence dropping off in another third and the final third of recruits did not adopt the intervention at all.
- Barriers and facilitators were explored in interviews and a focus group with app users.
- Important barriers to adherence were interface problems, orientation towards technology, lack of support from others and competing priorities.
- Important facilitators of adherence were technology receptiveness, having support from others, tailoring to individual needs and being more recently diagnosed.
- These barriers and facilitators are now informing further development of the ASICA intervention.

## Supplementary Material

llad279_Supplementary_Data

## Data Availability

The data underlying this article are available upon request from the corresponding author.
