# Supplementary material for: Barriers and facilitators of adherence to the use of ASICA, a digital app designed to support people previously treated for melanoma: concise report of a qualitative study
Source: Clin Exp Dermatol. 2023 Aug 23;48(12):1358–60. doi: 10.1093/ced/llad279 (PMC10651757; doi:10.1093/ced/llad279)
Supplement: llad279_Supplementary_Data [file llad279_supplementary_data.docx]

Appendix S1. Selected illustrative participant quotes.

| **Technology** |
| --- |
| " I hate that ruddy bit of kit I’m supposed to sort of keep in communication with you, it’s a Samsung thing, I’m an Apple man, so I hate the damn thing, so that gets me frustrated, because I don’t understand" (86-year-old male, secondary analysis)  Shall we say, from the initial meeting I went and the bit of training, there was a couple of people who were unsure as soon as they…as soon as the minipad came out, and the call was they would never use one (69-year-old male, primary interviews)  “No, it's [AI] just such a horrific thing. You don't want anything to rely on a database and questions and computer collate things”. (66-year-old female, primary interviews)  “Yes, absolutely, I wouldn’t have any issues with submissions I made seeing being run through to that sort of simulation or that process”. (44-year-old female, primary interviews)  “Yeah, but I practiced taking a photograph and then how it would upload, and it all seemed really easy because usually attaching photographs to things isn't something I would usually get right the first time”. (55-year-old-female, primary interviews)  “You know you’ve got a smartphone for a starter, and without a smartphone, without ….you’re using a smartphone, people will stop or not even start it.” (60-year-old male, primary interviews)  “I had to go through the whole tutorial again, which was you could just see it there, you couldn't, you couldn't Fast forward it because I knew what to do”. (42-year-old male, primary interviews)  “Video probably would be quite good because you could then go back and watch it again and again. If you can't do it, probably and, uh, at least with a leaflet as well, but if you get something and you don't want to read the whole, watch the whole video you can read the leaflet. And then maybe for people that are not that comfortable, have a group session.” (66-year-old, female, primary interviews) |
| **Role of Others** |
| It was probably having somebody to motivate you and be along with you with it. (55-year-old-female, primary interviews)  “I thought it was very good, but I, like myself, that I found it easier to involve family, my wife, ‘cause my back is this area that you don’t see very often, so my wife helped me with the back of my legs, and the soles of my feet, stuff like that… I needed that; I couldn’t manage on my own”. (76-year-old male, focus group)  “I wanted to be part of the trial and helping other people” (58-year-old male, secondary analysis)  “Aye, I suppose I'm just… asking them how they are or how they're finding the process. I just think something… human interaction rather than just a simple reminder of it.” (50-year-old female, primary interviews) |
| **Tailoring** |
| “Additional information about the trial , any developments, any new information or new findings. It’s just a [inaudible] ….bring it to life a bit more.  "Well, it’s good because if I didn’t have it, I honestly would, I mean every time it pings I go,  ‘God, is that another month? [laughs] (78-year-old, female, secondary interviews)  “And then there comes a point in recovery from a cancer that you, you feel more confident, and you don't want to keep being reminded of that all the time every month. (56-year-old female, primary interviews) |
| **Disease Journey** |
| Because it possibly found that there was no changes and so they just though, och I haven't had any changes in the last few months, so it's not going to change now. So, they just stopped”. (65-year-old female, primary interviews)  “I was scared it came back. That that simple I just, you know, I was scared I had such a shock to get”. (66-year-old female, primary interviews) |
| **Competing Priorities** |
| “It just, it could just be life fortunately. When life throws you some trouble, you you forget” (43-year-old female, primary interviews)  “Maybe something happened to them that they couldn't do it because something else became more important the other part of our health became more important and so they didn't bother looking at it” (65-year-old female, primary interviews)  “It's so easy for them to check the 3-4 pictures you know if you have 20, is getting very time consuming and you know, probably losing the interest” (42-year-old male, primary interviews) |
